# Improving Gemcitabine Sensitivity in Pancreatic Cancer Cells by Restoring miRNA-217 Levels

**DOI:** 10.3390/biom11050639

**Published:** 2021-04-26

**Authors:** Concetta Panebianco, Nadia Trivieri, Annacandida Villani, Fulvia Terracciano, Tiziana Pia Latiano, Adele Potenza, Francesco Perri, Elena Binda, Valerio Pazienza

**Affiliations:** 1 Gastroenterology Unit, Fondazione IRCCS Casa Sollievo della Sofferenza, 71013 San Giovanni Rotondo, Italy; panebianco.c@gmail.com (C.P.); a.villani@operapadrepio.it (A.V.); terracciano74@hotmail.com (F.T.); f.perri@operapadrepio.it (F.P.); 2 Cancer Stem Cell Unit, Institute for Stem-Cell Biology, Regenerative Medicine and Innovative Therapies (ISBReMIT), Fondazione IRCCS Casa Sollievo della Sofferenza, 71013 San Giovanni Rotondo, Italy; n.trivieri@css-mendel.it; 3 Oncology Unit Fondazione IRCCS Casa Sollievo della Sofferenza, 71013 San Giovanni Rotondo, Italy; latianotiziana@gmail.com; 4 Dietetic and Clinical Nutrition Unit, Fondazione IRCCS Casa Sollievo della Sofferenza, 71013 San Giovanni Rotondo, Italy; a.potenza@operapadrepio.it

**Keywords:** miRNA, pancreatic cancer, chemoresistance

## Abstract

Chemoresistance is a major problem in the therapeutic management of pancreatic cancer, concurring to poor clinical outcome. A number of mechanisms have been proposed to explain resistance to gemcitabine, a standard of care for this malignancy, among which is included aberrant miRNA expression. In the current study, we investigated the role of miR-217, which is strongly down-regulated in cancerous, compared to normal, pancreatic tissues or cells, in sensitizing human pancreatic cancer cell lines to this drug. The low expression of miR-217 in pancreatic cancer patients was confirmed in two gene expression datasets (GSE41372 and GSE60980), and the prognostic value of two target genes (ANLN and TRPS1), was estimated on clinical data from the Tumor Cancer Genome Atlas (TCGA). Transfecting miR-217 mimic in pancreatic cancer cells reduced viability, enhanced apoptosis, and affected cell cycle by promoting a S phase arrest in gemcitabine-treated cells. Moreover, in drug-exposed cells subjected to miR-217 forced expression, a down-regulation for several genes involved in cancer drug resistance was observed, many of which are cell cycle regulators, such as *CCND1*, *CCNE1*, *CDK2*, *CDKN1A*, *CDKN1B*, while others, such as *ARNT, BRCA1, BRCA2, ELK1, EGFR, ERBB4,* and *RARA* are involved in proliferation and cell cycle progression. Our results support the notion that miR-217 enhances pancreatic cancer sensitivity to gemcitabine, mainly impairing cell cycle progression.

## 1. Introduction

The seventh most common cause of death for cancer worldwide and third in developed countries, pancreatic ductal adenocarcinoma (PDAC) is usually a fatal disease [1], with a 5 year survival rate of around 9% from the time of diagnosis [2]. Due to the lack of early specific symptoms and late diagnosis, surgery is a valid curative option in only 15 to 20% of PDAC carriers [3], while for the remaining patients gemcitabine-based chemotherapy represents the mainstay treatment [4]. Gemcitabine is a synthetic nucleoside analog in which the hydrogen atoms on the 2′ carbon of deoxycytidine are replaced by fluorine atoms. The drug enters the cells through two different kinds of membrane carriers, called equilibrative and concentrative nucleotide transporters (hENTs and hCNTs) respectively, even though the most of its uptake is mediated by hENT1. Within the cell, gemcitabine is activated through three steps of phosphorylation, the first of which, mediated by the enzyme deoxycytidine kinase (dCK), is the rate-limiting one. The main mechanism of action of gemcitabine is to discontinue the DNA synthesis: once incorporated, it then inhibits chain elongation. Moreover, the diphosphorylated form of gemcitabine inhibits the enzyme ribonucleotide reductase subunit M1 or M2 (RRM1/RRM2), thus reducing the cellular pool of deoxyribonucleotides and making gemcitabine more likely to be incorporated into DNA. An additional mode of action of gemcitabine is the induction of apoptosis [5]. Despite being the cornerstone of PDAC therapy, however, the overall benefits provided by gemcitabine are limited. Intrinsic or acquired mechanisms of drug resistance, indeed, represent a major challenge for its effectiveness [6,7], rendering the clinical outcome mostly unsatisfactory. For this reason, developing strategies to overcome this drawback would be of remarkable clinical significance.

A number of potential mechanisms underlying gemcitabine resistance have been identified and extensively reviewed, among which is aberrant expression of microRNAs (miRNAs) [6,7]. The latter are small non-coding RNAs of about 20–22 nucleotides in length regulating gene expression at the post-transcriptional level. miRNAs are classically known to bind to a complementary sequence on the 3′-UTR region of mRNAs, causing its degradation, inhibiting translation into proteins, or both [8]. A number of studies, however, have shown that miRNAs can also bind to 5′-UTR or to the ORF [9,10,11,12,13], and that they can even up-regulate target gene expression [14,15,16]. By modulating gene expression, miRNAs influence several biological processes, among which are development, differentiation, proliferation, and apoptosis [17]; when their expression is dysregulated, alterations of such processes may trigger carcinogenesis [18]. Beside a plethora of studies documenting the role of miRNAs in cancer onset and progression, many reports have also demonstrated that miRNAs may impact on the outcome of anticancer therapies, either by enhancing drug efficacy or by inducing chemoresistance [19,20].

As reviewed by other groups, a conspicuous number of miRNAs have been found up- or downregulated in PDAC and some have been associated with response to chemotherapy [21,22,23]. We focused our attention on miR-217, which was shown to be down-regulated in PDAC tissues and cell lines compared to their noncancerous counterpart [24,25,26,27,28,29,30] and to function as a tumor suppressor miRNA in PDAC [25,26,29,30]. No previous association between miR-217 and PDAC chemoresistance has been demonstrated, nevertheless a role for miR-217 in enhancing chemosensitivity of other types of cancers has been described [31,32,33,34,35,36]. Guo et al. found that miR-217 over-expression in lung cancer cells significantly decreases cell viability upon cisplatin treatment in a dose-dependent and time-dependent manner [31]. Similarly, miR-217 was found to sensitize cervical cancer cells to cisplatin, as demonstrated by reduced cell viability [36]. Xiao et al. showed that ectopic expression of miR-217 in acute myeloid leukemia cells increases chemosensitivity to doxorubicin, since it increases the growth inhibition rate and enhances apoptosis [34]. Similarly, in chronic myeloid leukemia cells, miR-217 over-expression was able to reverse resistance to the tyrosine kinase inhibitors imatinib [33] and dasatinib [35]. Finally, miR-217 over-expression reversed resistance to paclitaxel and doxorubicin in gastric cancer cells [32]. Based on these observations, the aim of the present study was to investigate whether miR-217 modulates the sensitivity to gemcitabine in two PDAC cell lines (with different genetic background, such as BxPC-3 and KRAS mutated PANC-1 cells) given the still disappointing effectiveness of this chemotherapeutic agent. Moreover, the novelty of our study relies also on the bioinformatic approach, which paves the way to the discovery of new pathways/targets involved in pancreatic cancer tumorigenesis. Our results confirm that miR-217 is among the downregulated miRNAs in human pancreatic cancer compared to normal tissue, it is poorly expressed in BxPC-3 and PANC-1 cancer cell lines relative to pancreatic control cells and provide evidence that forcing miR-217 expression ameliorates response to gemcitabine, as demonstrated by a decrease in cell count and viability, an increase in cell cycle arrest, and a higher percentage of late-apoptotic cells.

## 2. Materials and Methods

### 2.1. Microarray Dataset Investigation and Detection of Differentially Expressed miRNAs and mRNAs

In silico analysis was performed to identify miR-217 target genes. In order to find proper miRNA and mRNA dataset, a systematic search in Gene Expression Ominibus (GEO) dataset (https://www.ncbi.nlm.nih.gov/geo, accessed on 22 March 2021) was conducted (Barret. et al., 2012). By using the keywords human miRNA and mRNA dataset in Pancreatic Cancer, several datasets were found. Two of these (GSE41372 and GSE60980), containing either miRNA or mRNA arrays, were then selected. Differentially expressed miRNAs (DE-miRNAs)/genes (DEGs) were screened with GEO2R in GEO dataset, by which two groups of samples are compared using the GEOquery and limma R packages from the Bioconductor project. Normalization was carried out using RMA algorithm. The micro-arrays gene expression profiles of the two datasets were compared to the normal groups of each dataset, separately. DE-miRNAs and DE-mRNAs were called on a minimum log2 fold change of 1.3, and the false discovery rate (FDR) was controlled by adjusting *p* value with the Benjamini–Hochberg algorithm (*q* < 0.05). All significant DE-miRNAs and DEGs were depicted in a volcano plot generated using GraphPad Prism software v.8.0. miRNA target genes were identified by Ingenuity Pathway Analysis Software (IPA; QIAGEN) microRNA Target Filter, that includes filtering tools sorting microRNA targets and allowing the examination of microRNA-mRNA pairing. Lists of differentially expressed miRNAs and mRNAs from each dataset were used to find target genes.

### 2.2. Survival and Statistical Analysis

To evaluate the relationship between microRNA target genes and patients’ outcome, mRNA expression data and corresponding clinical information for Pancreatic Adenocarcinoma (PAAD) dataset of the TCGA were downloaded from Morpheus (Broad Institute, Cambridge, MA; https://software.broadinstitute.org/morpheus/ (accessed on 22 March 2021). At first, 183 patients with available mRNA Seq data from PAAD dataset and overall survival (OS) information were selected. Then, as described in Peran et al. [37], samples of various origin and (pseudo)- normal samples were excluded. For the 150 PDAC samples, optimal cutoff between high and low miRNA expression groups were determined through the R package “survminer”. Survival curves were evaluated using GraphPad Prism v.7.0 software by Kaplan–Meier method and overall comparisons performed by log-rank test. *p*-values < 0.05 were considered significant.

### 2.3. Ingenuity Pathway Analysis

The function “Core Analysis” included in the Ingenuity Pathway Analysis software (IPA; QIAGEN, http://www.ingenuity.com/ (accessed on 29 January 2021)) was used to perform an in silico analysis of the diseases, biological functions and canonical pathways in which miR-217 and its target mRNAs are involved.

### 2.4. Cell Culture, miRNA Transfection, and Gemcitabine Treatment

The normal Human Pancreatic Duct Epithelial cell line (HPDE) was cultured in Keratinocyte serum-free medium supplemented with epidermal growth factor (5 ng/mL) and bovine pituitary extract (50 µg/mL) (Thermo Fisher Scientific, Milan, Italy) in 5% CO_2_ atmosphere at 37 °C. The human pancreatic cancer cell lines BxPC-3 and PANC-1 were cultured in RPMI medium supplemented with 10% fetal bovine serum, 100 U/mL penicillin and 100 µg/mL streptomycin (Thermo Fisher Scientific, Milan, Italy) in 5% CO_2_ atmosphere at 37 °C. BxPC-3 and PANC-1 cells were seeded in 12-well plates and were reverse-transfected with miR-217 mimic and the AllStars-Negative Control (AllStars-NC) siRNA or were mock-transfected using the HiPerFect Transfection Reagent (Qiagen, Milan, Italy) in serum free medium, according to the manufacturer’s instructions. The following day, cells were treated or not with 1µM gemcitabine (Sigma-Aldrich, Milan, Italy) for 48 h.

### 2.5. miRNA Isolation from Cells, Reverse-Transcription, and Quantification

Total RNA (including miRNAs) was isolated from cultured cells using the miRNeasy Mini Kit (Qiagen), according to the manufacturer’s instructions. miRNAs were reverse transcribed using the TaqMan MicroRNA Reverse Transcription kit (Thermo Fisher Scientific) and primers for hs-miR-217 and RNU6B (TaqMan MicroRNA Assay, Thermo Fisher Scientific). Expression of miR-217 relative to RNU6B was determined using the TaqMan Universal PCR Master Mix (Thermo Fisher Scientific). Reactions were run on a 7900HT Real-Time PCR System (Applied Biosystems, Foster City, CA, USA) and all samples were assayed in triplicate. Optical data obtained were analyzed using the default and variable parameters available in the SDS software package (version 2.4; Applied Biosystems, Foster City, CA, USA). Relative expressions were calculated through the 2^(−ΔΔCt)^ method.

### 2.6. Count and Viability Assay

Cells were counted and viability assessed by flow cytometric analysis using the Muse Count & Viability assay kit (Merck Millipore, Milan, Italy). 48 h after gemcitabine treatment, cells were detached and 50 µL of cell suspension were incubated with 450 µL of the Count & Viability reagent for 5 min, according to the manufacturer’s protocol, before loading on the Muse Cell Analyzer (Merck Millipore, Milan, Italy).

### 2.7. Apoptosis Assay

The quantification of live, early apoptotic, late apoptotic, and dead cells upon the different experimental conditions was performed by means of the Muse Annexin V and Dead Cell Assay kit (Merck Millipore, Milan Italy), at the Muse Cell analyzer, accordingly to the supplier’s instructions. Briefly, a cell suspension was prepared and 100 µL were incubated with an equal volume of Muse Annexin V & Dead Cell Reagent for 20 min in the dark, before loading on the Muse Cell Analyzer.

### 2.8. Cell Cycle Analysis

The effects of the miR-217 over-expression and gemcitabine treatment on cell cycle distribution of overnight serum deprived synchronized pancreatic cancer cells were assessed by flow cytometric analysis using the Muse Cell Cycle kit (Merck Millipore, Milan Italy) at the Muse Cell Analyzer, as previously described [38,39].

### 2.9. Cancer Drug Resistance Gene Expression

A commercially available array (Human RT^2^-Profiler, Qiagen) was used to assess the expression of 84 genes involved in cancer drug resistance in BxPC-3 and PANC-1 cells mock-transfected and miR-217-transfected treated or not with gemcitabine. qRT-PCR was performed on a 7900HT Fast Real-Time PCR device (Applied Biosystems, Foster City, CA, USA). Optical data obtained were analyzed using the SDS software package (version 2.3; Applied Biosystems, Foster City, CA, USA). Gene expression was normalized to that of the housekeeping gene ACTB.

### 2.10. Statistical Analysis

Data from qRT-PCR and Muse assays are expressed as mean ± SD. Comparisons were made using Student’s *t*-test, one-way ANOVA, and two-way ANOVA followed by a post hoc Tukey’s multiple comparisons test when appropriate. Differences were considered as statistically significant when *p* < 0.05 (*) or *p* < 0.01 (**) or *p* < 0.001 (***).

## 3. Results

### 3.1. Differential miRNAs and miRNAs Target Genes’ Expression in Pancreatic Cancer and Normal Tissue

**Figure 1:**
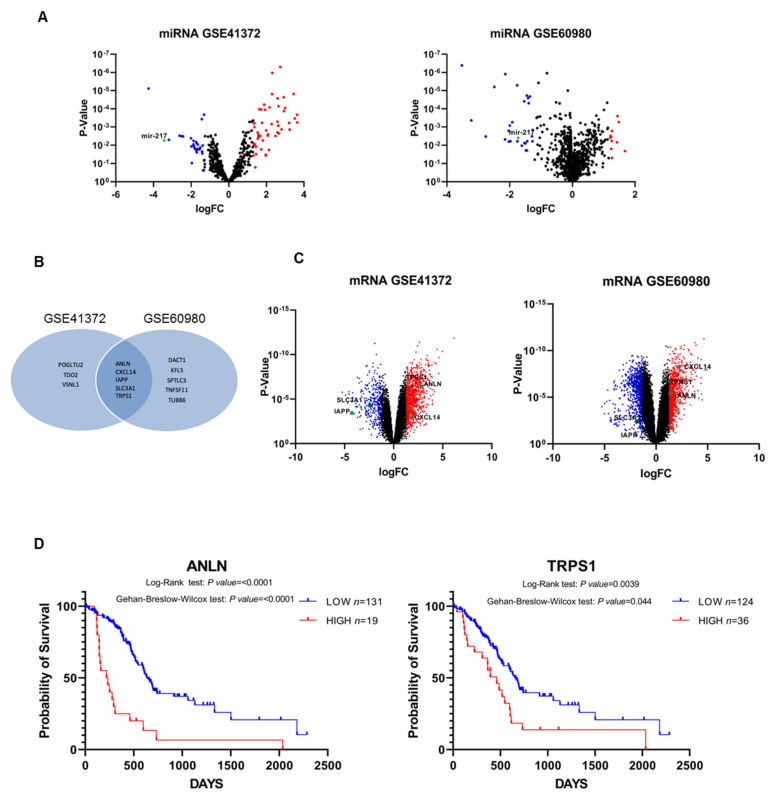
Volcano plot representation of the differentially expressed miRNAs between pancreatic cancer and normal tissue in GSE41372 and GSE60980 datasets (**A**). A VENN diagram showing the shared miR-217 target genes highly predicted and experimentally observed filtered from GSE41372 and GSE60980 datasets (**B**). Volcano plot representation of the differentially expressed mRNAs between pancreatic cancer and normal tissue in GSE41372 and GSE60980 datasets (**C**). Prognostic value of target mRNAs in pancreatic cancer versus normal tissues. Kaplan–Meier survival curves for 150 pancreatic cancer patients from TCGA dataset revealing that low expression levels of ANLN and TRPS1 are associated with a better probability of survival. Statistical differences were analyzed using the log-rank test (**D**).

As a first approach we examined two independent datasets available at the GEO repository under the accession numbers GSE41372 and GSE60980, in order to screen differentially expressed miRNAs and mRNAs between pancreatic cancer and normal tissue. This research revealed 715 miRNAs and 1305 mRNAs for dataset GSE41372, whereas 1348 miRNAs and 1732 mRNAs for GSE60980. As shown in Volcano plots in Figure 1A, in both datasets miR-217 was confirmed down-regulated in pancreatic cancer with respect to normal tissue. In addition, miRNA targets highly predicted and experimentally observed were filtered from the two datasets (Tables S1 and S2) and compared with each other. Five mRNAs were shared between the two datasets (Figure 1B), with SLC3A1 and IAPP down-regulated whereas CXCL14, ANLN, and TPRS1 up-regulated in pancreatic cancer (Figure 1C). The latter two genes were also found to have a prognostic value in pancreatic cancer patients, since Kaplan–Meier survival analysis showed that subjects with low expression of ANLN and TRPS1 were associated with a longer survival than those with high levels (*p* < 0.0001 and *p* = 0.0039, respectively, by log-rank Mantel–Cox test) (Figure 1D).

### 3.2. Disease and Biological Functions and Canonical Pathway Analysis

**Figure 2:**
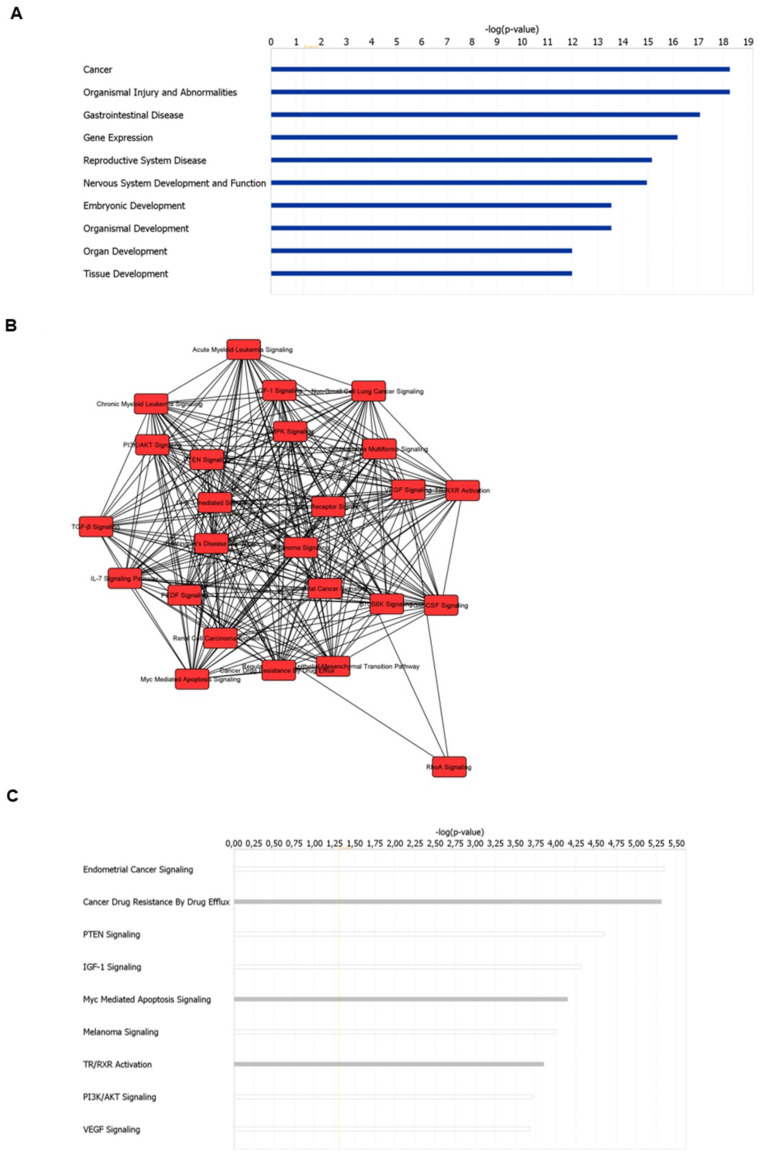
The top ten enriched diseases and biological functions of miR-217 according to IPA Core Analysis (**A**). The network of canonical pathways (**B**) and the most represented canonical pathways (**C**) of miR-217 and its target genes, according to IPA Core Analysis. Grey bars represent pathways with no activity predicted due to a lack of information in the Ingenuity Pathway Knowledge Base (IPKB), despite highly significant association between genes and pathway. White bars represent pathways with z-score of 0, pointing out that differentially expressed genes did not indicate an evident prediction of activation or inhibition.

A bioinformatic prediction of the disease and biological functions related to miR-217 placed cancer and gastrointestinal disease as, respectively, the first and the third top diseases in which miR-217 is involved (Figure 2A and Table S3), thus supporting the potential role of this miRNA in pancreatic cancer. Moreover, an analysis of the canonical pathways associated to miR-217 and its target mRNAs showed that miR-217 is strongly involved in mechanisms of cancer drug resistance (Figure 2B,C and Table S4).

### 3.3. miR-217 Over-Expression in PDAC Cell Lines

It is known that miRNA deregulation is an emerging cause of chemoresistance in different types of cancers [19,20], and specifically miR-217, whose expression levels are low in PDAC tissues and cell lines [24,25,26,27,28,29,30], is inversely correlated with resistance to different chemotherapeutics [40,41]. Interestingly, miR-217 was found to enhance chemosensitivity of lung cancer cells to cisplatin [31], of acute myeloid leukemia cells to doxorubicin [34], of chronic myeloid leukemia cells to imatinib [33], and dasatinib [35], of cervical cancer cells to cisplatin [36], and of gastric cancer cells to paclitaxel and doxorubicin [32]. In the light of all these observations, we sought to assess whether overexpressing miR-217 in PDAC cell lines could enhance sensitivity to gemcitabine.

**Figure 3:**
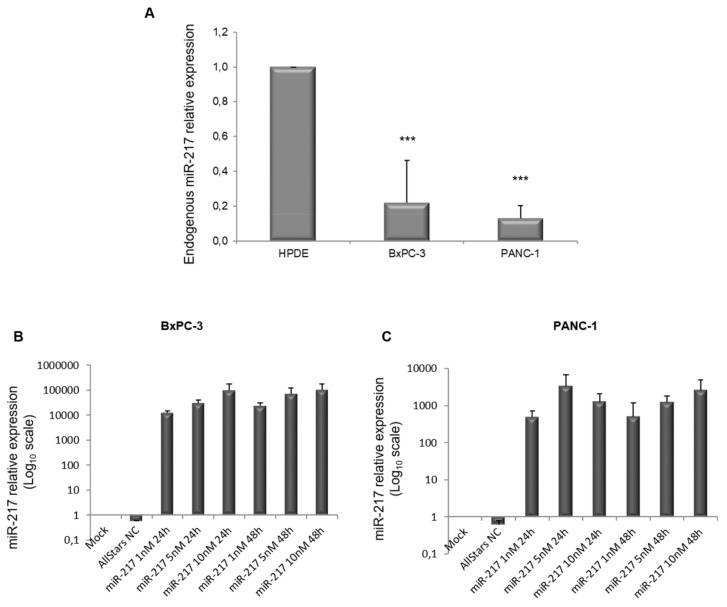
miR-217 endogenous expression in control HPDE cells and in BxPC-3 and PANC-1 PDAC cell lines. The relative miR-217 expression in the PDAC cell lines was much lower than that in the control cells (**A**). Dose-response (1 nM, 5 nM, and 10 nM) and time-course (24 h and 48 h) of miR-217 mimic transfection in BxPC-3 (**B**) and PANC-1 (**C**) cells (Log_10_ scale on the *y*-axis). *** *p*-values < 0.001.

First of all, we performed qRT-PCR to assess the endogenous levels of miR-217 in the PDAC BxPC-3 and PANC-1 cell lines, compared to the control HPDE cells. In agreement with previous studies [26,30], miR-217 expression was markedly lower in PDAC cell lines, with respect to normal cells (Figure 3A).

We next performed a dose-response (1 nM, 5 nM, and 10 nM) and time-course (24 h and 48 h) of miR-217 transfection in BxPC-3 and PANC-1 cells (Figure 3B,C, respectively) to establish miRNA over-expression. In all the conditions tested, a considerable over-expression was achieved. We set 5 nM for 48 h as the experimental condition from there on.

### 3.4. miR-217 Affects Count and Viability of PDAC Cells Upon Gemcitabine Treatment

**Figure 4:**
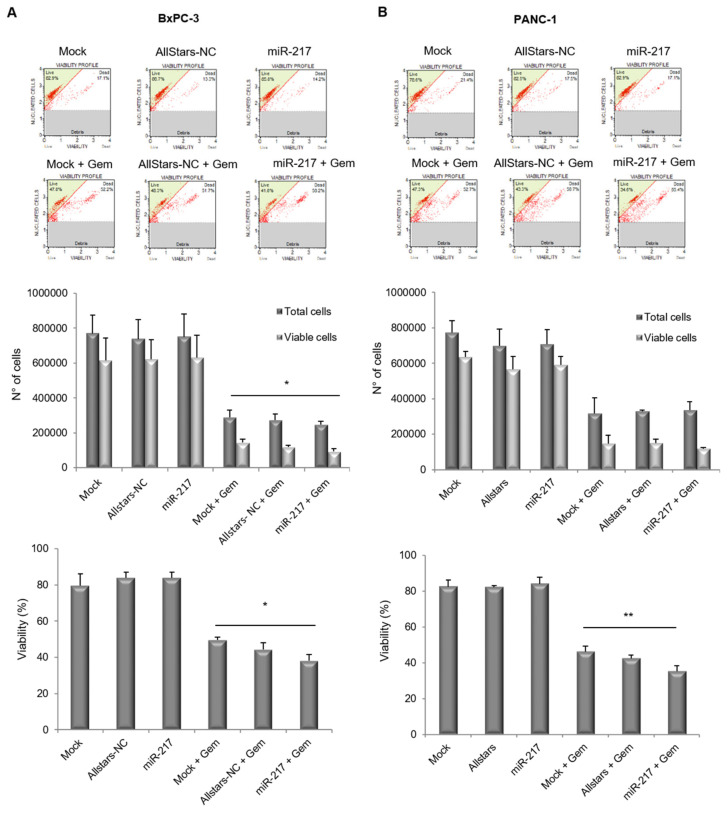
Flow cytometric analysis of cell count and viability performed by using Muse Cell Analyzer in mock-transfected, AllStars-transfected and miR-217-transfected BxPC-3 (**A**) and PANC-1 (**B**) cells alone, or upon gemcitabine treatment. Bar charts show the quantitative measurements expressed as mean ± SD of three independent experiments. Statistical differences were calculated by two-way ANOVA for the count of total and viable, and by one-way ANOVA for percentage of viability, respectively. *p* < 0.05 (*) and *p* < 0.01 (**).

In order to assess whether miR-217 affected cell growth and viability of PDAC cells, BxPC-3 and PANC-1 mock-transfected or transfected with either the AllStars-NC or the miR-217 mimic, were treated or not with gemcitabine for 48 h. Cells were then counted and viability was evaluated at the Muse Cell Analyzer (Figure 4). Cells transfected with the negative control did not show any significant change, compared to their mock-transfected counterpart. Over-expression of miR-217 in the absence of gemcitabine treatment had no effect on cell growth and viability in either of the two cell lines. Interestingly, in BxPC-3 cells, two-way ANOVA for Total cells and Viable cells upon gemcitabine treatment revealed statistical significance either for the variable “cell count” (i.e., total cells and viable cells *p* < 0.0001) and for the variable “experimental condition” (i.e., Mock + Gem, Allstars-NC + Gem and miR-217 + Gem conditions *p* = 0.0303). Concerning PANC-1 cells, statistical significance was only detected for the variable “cell count” (i.e., total cells and viable cells *p* < 0.0001) but not for the variable “experimental condition”. Moreover, a one-way ANOVA was performed as for percentage of viability, showing statistically significant differences upon gemcitabine treatment for both BxPC-3 (*p* = 0.0124) and PANC-1 (*p* = 0.0093) cells.

### 3.5. miR-217 Affects Apoptosis of PDAC Cells upon Gemcitabine Treatment

**Figure 5:**
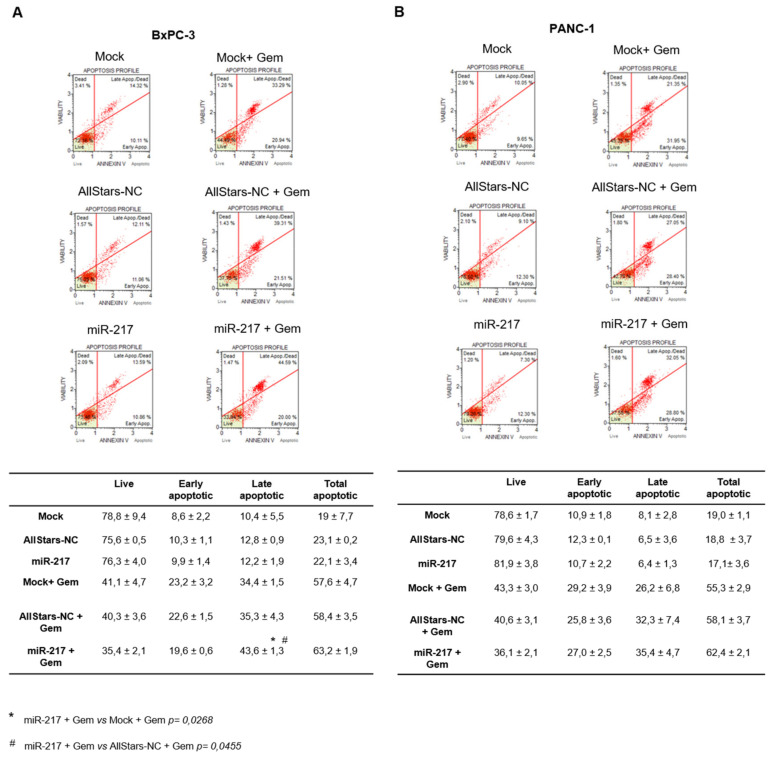
Flow cytometric analysis of apoptosis performed by using Muse Cell Analyzer in mock-transfected, AllStars-transfected, and miR-217-transfected BxPC-3 (**A**) and PANC-1 (**B**) cells alone, or upon gemcitabine treatment. Tables show the quantitative measurements expressed as mean ± SD of three independent experiments. Statistical differences were calculated by two-way ANOVA, followed by post hoc Tukey’s multiple comparisons test.

To find out whether induction apoptosis could explain the decline in cell viability observed in miR-217-transfected cells upon gemcitabine treatment, apoptosis was assayed at the Muse Cell analyzer. Again, no difference in apoptosis was found following miR-217 mimic transfection, in either BxPC-3 and PANC-1 cells not receiving gemcitabine. Conversely, some differences emerged upon gemcitabine treatment. As for BxPC-3, by performing two-way ANOVA the variable “vitality state” (i.e., live, early apoptotic, late apoptotic, or total apoptotic *p* < 0.0001) and the interaction between the two variables (*p* < 0.0385) were found significant. Since a main effect was found significant, a post hoc Tukey’s multiple comparisons test was performed. Significant results were obtained for late apoptotic cells when comparing Mock + Gem vs. miR-217 + Gem (adj *p* value = 0.0268) and AllStars-NC + Gem vs. miR-217 + Gem (adj *p* value= 0.0455) (Figure 5A). As for PANC-1 cells, only the variable “vitality state” resulted significant (*p* < 0.0001), but no other significance emerged from the Tukey’s multiple comparisons test (Figure 5B).

### 3.6. miR-217 Enhances Gemcitabine Effects on Cell Cycle of PDAC Cells

We next analyzed the cell cycle of PDAC cells under the different experimental conditions. In both cell lines gemcitabine treatment, irrespective of the transfection, increased the proportion of cells in S phase, which is a well-documented mechanism of action of this drug [42,43,44,45].

**Figure 6:**
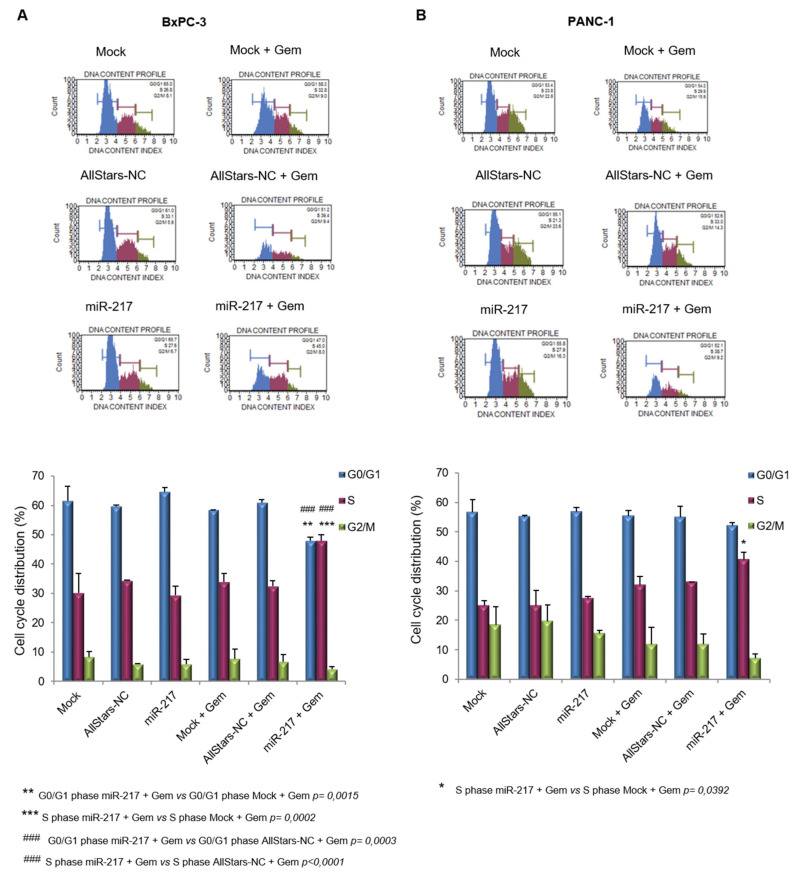
Flow cytometric analysis of cell count and viability performed by using Muse Cell Analyzer in mock-transfected, AllStars-transfected, and miR-217-transfected BxPC-3 (**A**) and PANC-1 (**B**) cells alone, or upon gemcitabine treatment. Bar charts show the quantitative measurements expressed as mean ± SD of three independent experiments. Statistical differences were calculated by two-way ANOVA, followed by post hoc Tukey’s multiple comparisons test.

When two-way ANOVA was performed in BxPC-3 cells, the variable “cell cycle phase” (i.e., G0/G1, S or G2/M phase *p* < 0.0001) and the interaction between the two variables (*p* < 0.0001) were significant. The post hoc Tukey’s multiple comparisons test showed significant results when comparing Mock + Gem vs. miR-217 + Gem (adj *p* value = 0.0015) and AllStars-NC + Gem vs. miR-217 + Gem (adj *p* value = 0.0003) in G0/G1 phase and for the comparisons Mock + Gem vs. miR-217 + Gem (adj *p* value = 0.0002) and AllStars-NC + Gem vs. miR-217 + Gem (adj *p* value < 0.0001) in S phase (Figure 6A). Concerning the two-way ANOVA analysis in PANC-1 cells, the variable “cell cycle phase” (i.e., G0/G1, S, or G2/M phase *p* < 0.0001) and the interaction between the two variables (*p* = 0.0435) were significant. The only significant result was obtained for the comparison Mock + Gem vs. miR-217 + Gem (adj *p* value = 0.0015) in S phase (Figure 6B) by the post hoc Tukey’s multiple comparisons test.

### 3.7. Gemcitabine Treatment and miR-217 Over-Expression Affect Cancer Drug Resistance Gene Expression

**Figure 7:**
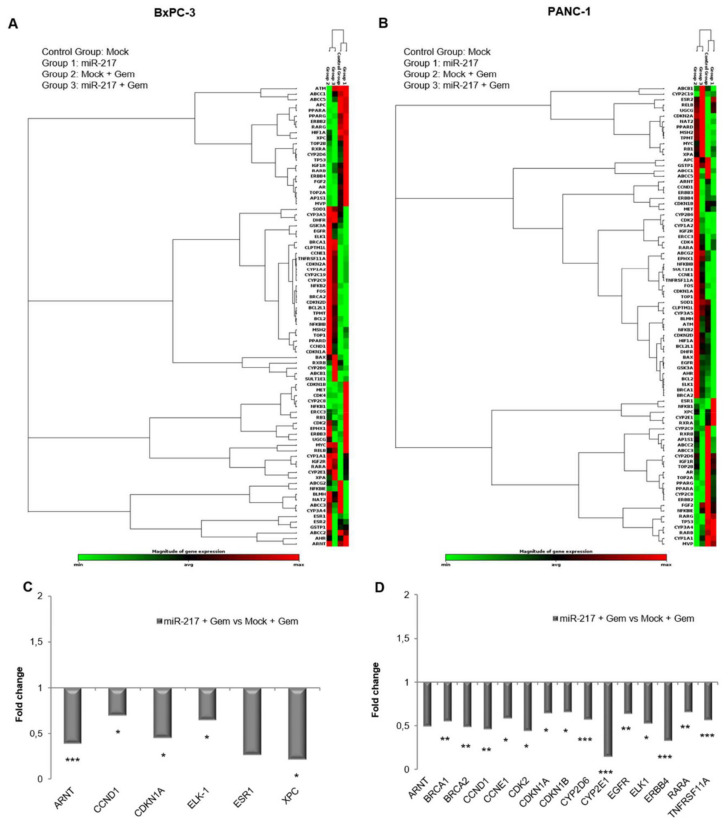
Clustergram representation of cancer drug resistance genes expression in mock-transfected and miR-217-transfected BxPC-3 (**A**) and PANC-1 (**B**) cells alone, or upon gemcitabine treatment. Fold change of cancer drug resistance gene expression in miR-217-transfected versus mock-transfected BxPC-3 (**C**) and PANC-1 (**D**) cells. The plots show the means of two independent experiments for each cell lines. *p* < 0.05 (*) or *p* < 0.01 (**) or *p* < 0.001 (***).

To get more insight into the molecular mechanisms potentially underlying the gemcitabine sensitization upon miR-217 transfection, the expression of 84 genes involved in cancer drug resistance was measured by using a commercially available gene array. Since our previous results have shown that no change was induced by AllStars-NC transfection compared to mock, gene expression was only investigated in mock- and miR-217-transfected cells treated or not with gemcitabine. Chemotherapy administration had the greatest impact on cancer drug resistance gene expression in both cell lines, as demonstrated by the clustergrams in Figure 7A,B. Focusing on the gemcitabine-sensitizing effect on miR-217 over-expression and comparing miR-217 + Gem versus Mock + Gem condition, using a 1.5-fold cutoff difference in mRNA expression, a number of genes resulted dysregulated above all in PANC-1 cells, with some overlaps between the two cell lines. Concerning BxPC-3, statistically significant down-regulation was found for ARNT, CCND1, CDKN1A, ELK1, and XPC genes, encoding Aryl Hydrocarbon Receptor Nuclear Translocator (also known as HIF-1-Beta), Cyclin D1, Cyclin-dependent Kinase Inhibitor 1A (also called p21), ETS-Like Gene 1, and Xeroderma pigmentosum, complementation group C, respectively. An interesting decreasing trend was obtained for ESR1 encoding estrogen receptor 1 (Figure 7C). ARNT tended to decrease in miR-217 + Gem versus Mock + Gem also in PANC-1 cells, which shared with BxPC-3 a significant decline in CCND1, CDKN1A, and ELK1. In addition, the expression of BRCA1 (Breast cancer 1), BRCA2 (Breast cancer 2), CCNE1 (Cyclin E1), CDK2 (Cyclin Dependent Kinase 2), CYP2D6 (Cytochrome P450 Family 2 Subfamily D Member 6), CYP2E1 (Cytochrome P450 Family 2 Subfamily E Member 1), EGFR (Epidermal Growth Factor Receptor, or ERBB1), ERBB4 (Erb-B2 Receptor Tyrosine Kinase 4), RARA (Retinoic Acid Receptor Alpha), and TNFRSF11A (TNF Receptor Superfamily Member 11a) genes also exhibited a statistically significant drop (Figure 7D).

**Figure 8:**
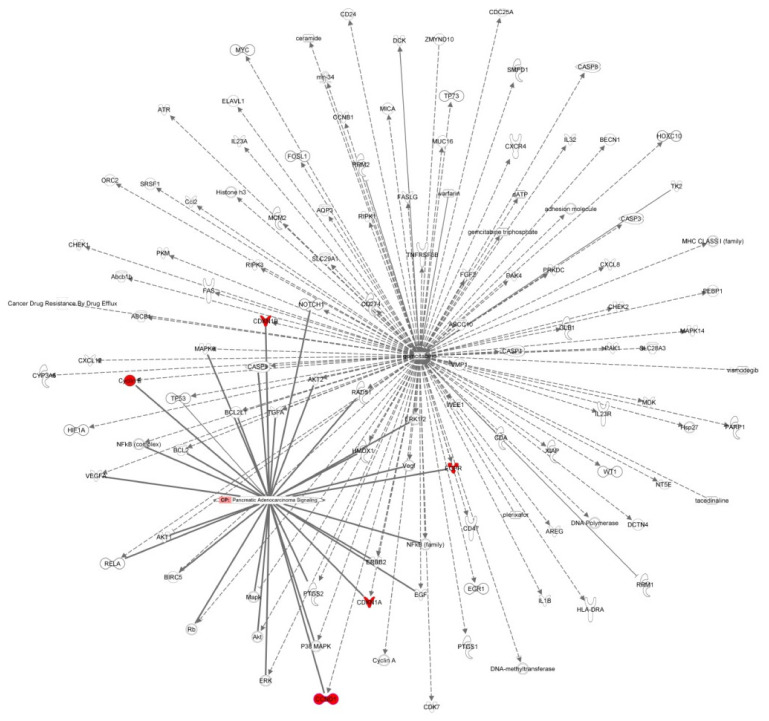
Interaction network between pancreatic adenocarcinoma signaling and gemcitabine pathway generated from IPA. Shared genes are red colored.

An IPA interaction network analysis revealed the genes CCND1, CDK1A, CDK1B, Cyclin E (also known as CCNE1), and EGFR at the interface between the gemcitabine pathway and the pancreatic cancer signaling (Figure 8). Interestingly, we found the expression of these genes to be affected by miR-217 overexpression, further supporting the key role of miR-217 in mediating gemcitabine efficacy.

## 4. Discussion

Gemcitabine is the first line therapy for patients with advanced unresectable PDAC, even though it produces only moderate clinical response since the majority of patients, despite an initial response to therapy, develop chemoresistance over time [6,7,46]. Multiple mechanisms of gemcitabine resistance have been discovered, including inefficient drug uptake, enzymatic drug inactivation, impairment of apoptosis, increased expression of drug efflux transporters, activation of epithelial-mesenchymal transition, stemness pathways and aberrant expression of miRNAs [6,7]. The latter aspect is gaining increasing attention in recent years, since more and more miRNAs are being found to either enhance or decrease sensitivity of cancer cells to different chemotherapeutics [19,20].

A survey of the literature concerning miRNAs associated with PDAC unveiled that miR-217 is aberrantly under-represented in cancerous tissues and cell lines compared to normal pancreas [24,25,26,27,28,29,30], which we further confirmed by examining two different published datasets. In order to confirm this data in our experimental model, we measured the endogenous levels of miR-217 in BxPC-3 and PANC-1 cell lines and compared to that of the HPDE control cells. As expected, both PDAC cell lines expressed much lower levels of this miRNA than the control.

It is known that miR-217 levels are inversely correlated with resistance to several chemotherapeutics [40,41] and that its ectopic expression increases sensitivity of lung and cervical cancer cells to cisplatin [31,36], of leukemia cells to doxorubicin, imatinib [33,34], and dasatinib [35], and of gastric cancer cells to doxorubicin and paclitaxel [32]. No evidence, however, has been produced so far concerning an association between miR-217 and PDAC chemoresistance nor between miR-217 and gemcitabine resistance. The current study provides insights into the role of miR-217 in the modulation of PDAC cell lines’ sensitivity to gemcitabine. Over-expression of the miR-217 mimic, combined with gemcitabine treatment, caused a significant drop in the viability of both cell lines. No difference was observed in the count of total cells. Given that miR-217 over-expression was previously found to promote apoptosis in PDAC cells [25,29] and apoptosis is among the mechanisms of action of gemcitabine, we checked whether the observed decrease in viability was attributable to an increase in cell death. The combination of miR-217 over-expression and gemcitabine treatment produced a significant increase in late apoptotic BxPC-3 cells and a trend not reaching statistical significance in PANC-1. Since the percentage of total apoptotic cells was not significantly modified, we speculate that miR-217 transfection could have speeded up apoptosis. As an alternative, co-treatment with miR-217 and gemcitabine could have induced necrotic, rather than apoptotic, cell death, since late apoptotic and necrotic cells share positivity to both Annexin V and propidium iodide staining.

An investigation of the cell cycle distribution of BxPC-3 and PANC-1 under the different experimental conditions uncovered that miR-217 over-expression cooperates with gemcitabine to increase accumulation of cells in the S phase. Many pharmacodynamic studies revealed that gemcitabine affects cell cycle by inducing arrest in S phase when used at low and moderate doses, while imposing cell cycle arrest in all phases at higher concentrations [42,43,44,47,48]. Gemcitabine, as a nucleoside analog, exerts its cytotoxicity on cells in S phase, that is why an initial low dose, which synchronizes cells in S phase, would sensitize them to further administrations [47].

Expression analysis of cancer drug resistance genes further confirmed the involvement of cell cycle regulators in miR-217-induced sensitivity to gemcitabine exposition, especially in PANC-1 cells, in which CCND1, CCNE1, CDK2, CDKN1A, and CDKN1B, all participating in the transit across S phase, resulted in down-regulation. Over-expression of Cyclin D1 was found to confer gemcitabine and cisplatin resistance to pancreatic cancer cell [49]; CCNE1, CDK2, and CDKN1A, previously reported to be up-regulated by gemcitabine as confirmed in our study, were also implied in chemotherapy resistance [50,51].

In addition, ARNT, BRCA1, BRCA2, ELK1, EGFR, ERBB4, and RARA are also known to take part in regulating cell cycle progression [52,53,54,55,56,57].

Overall, our results strongly suggest that cell cycle regulation is a major mechanism, through which, miR-217 sensitizes PDAC cells to gemcitabine action and support a therapeutic potential for miR-217 in enhancing PDAC sensitivity to this drug.

## Data Availability

Not applicable.

## Supplementary Materials

The following are available online at https://www.mdpi.com/article/10.3390/biom11050639/s1, Table S1. miR-217 target genes from GSE41372 dataset. Table S2. miR-217 target genes from GSE60980 dataset. Table S3. Ingenuity Pathway Analysis of diseases and biological functions of miR-217. Table S4. Ingenuity Pathway Analysis of canonical pathways of miR-217.
